# Gamma-Oryzanol Solid Dispersion Attenuates Dexamethasone-Induced Depressive-like Behaviors in Rats Associated with Calbindin-1 and Calcium Signaling

**DOI:** 10.3390/ijms27198721

**Published:** 2026-09-29

**Authors:** Siripaporn Kesyou, Waree Tiyaboonchai, Jureepon Roboon, Paweena Kaewman, Sawanya Charoenlappanit, Sittiruk Roytrakul, Vincent Van Waes, Gavin P Reynolds, Samur Thanoi, Sutisa Nudmamud-Thanoi

**Affiliations:** 1 Faculty of Medical Science, Medical Science Graduate Program, Naresuan University, Phitsanulok 65000, Thailand; siripapornk62@nu.ac.th; 2 INSERM, UMR 1322 LINC, Université Marie et Louis Pasteur, F-25000 Besançon, France; vincent.van_waes@univ-fcomte.fr; 3 Department of Pharmaceutical Technology, Faculty of Pharmaceutical Sciences, Naresuan University, Phitsanulok 65000, Thailand; wareet@nu.ac.th; 4 Frontier Research Cluster in Brain Health, Naresuan University, Phitsanulok 65000, Thailand; jureeponr@nu.ac.th (J.R.); paweenakae@nu.ac.th (P.K.); 5 Department of Anatomy, Faculty of Medical Science, Naresuan University, Phitsanulok 65000, Thailand; 6 Centre of Excellence in Medical Biotechnology, Faculty of Medical Science, Naresuan University, Phitsanulok 65000, Thailand; gavin.reynolds@shu.ac.uk; 7 National Centre for Genetic Engineering and Biotechnology, National Science and Technology Development Agency, Pathum Thani 12120, Thailand; sawanya.cha@ncr.nstda.or.th (S.C.); sittiruk@biotec.or.th (S.R.); 8 Biomolecular Sciences Research Centre, Sheffield Hallam University, Sheffield S1 1WB, UK; 9 School of Medical Sciences, University of Phayao, Phayao 56000, Thailand

**Keywords:** depression, gamma-oryzanol, behavioral tests, calcium signaling, Calbindin, antidepressant effect

## Abstract

Depression is associated with dysfunction of the hypothalamic–pituitary–adrenal axis, neurotransmitter imbalance, and calcium signaling deficits. Although gamma-oryzanol (GO) has shown antidepressant effects, its underlying molecular mechanism remains elusive, while its poor aqueous solubility limits its nutraceutical application. To overcome this obstacle, we successfully developed GO solid dispersion (GOSD) with enhanced water solubility for nutraceutical formulations. This study examined the antidepressant effect of GOSD in dexamethasone (Dx)-induced depressive-like behaviors in rats using behavioral tests, including the sucrose preference test and forced-swimming test (FST). The Dx group showed a reduction in sucrose consumption and mobility time in the FST compared with controls, both of which improved following GOSD administration. Proteomics revealed changes associated with the calcium signaling pathway. Calbindin (Calb1) expression was reduced in the Dx group, whereas GOSD administration restored Calb1 levels. Given the role of Calb1, a calcium-binding protein (CBP) involved in intracellular calcium buffering, reduced Calb1 expression in the Dx group may contribute to calcium signaling dysregulation. In contrast, GOSD can attenuate Calb1 levels, which potentially contribute to its antidepressant effects. These findings suggest that the antidepressant-like effects of GOSD may involve modulation of calcium homeostasis, supporting its potential as a complementary intervention for Dx-induced depressive-like behaviors.

## 1. Introduction

Major depressive disorder (MDD) is a common psychiatric condition and an important contributor to the worldwide burden of disease [1]. Its clinical presentation involves poor mood, cognition, sleep, appetite, energy, and self-perception [2].The pathological mechanisms of MDD are linked to stress, which is a common pathophysiological response that the body initiates in reaction to undesirable situations. Prolonged exposure to stress can lead to biochemical, physiological, and behavioral changes and has also been linked to alterations in brain function, particularly in the frontal cortex [3,4]. The connection between stress and depression is mediated by hypothalamic–pituitary–adrenal (HPA) axis hyperactivity, which increases cortisol levels and induces abnormal cortisol rhythms in patients with depression [5]. This dysregulation contributes to mood disturbances and cognitive impairments commonly observed in MDD. Based on clinical findings, animal models were investigated to gain deeper insight into the molecular consequences of stress and the fundamental neurobiological processes involved in depression. One widely used approach involves the administration of synthetic glucocorticoids such as dexamethasone (Dx). Although Dx is not endogenously produced in rodents or humans, it potently suppresses endogenous corticosterone levels via inhibition of the HPA axis, leading to neuroendocrine imbalance [6]. Prolonged exposure to Dx has been shown to induce depressive-like behaviors, making it a valuable model for studying stress-related mood disorders that can also induce symptoms of depression. Moreover, several experiments have shown that long-term treatment with Dx leads to behavioral deficits commonly associated with depression. These include increased immobility time in the forced swim test (FST) and tail suspension test (TST), as well as a reduction in sucrose consumption in the sucrose preference test, which reflects anhedonia, a core symptom of depression [7,8,9,10].

It has also been reported that chronic glucocorticoid exposure disrupts the homeostasis of several neurotransmitter systems, including serotonin, dopamine, glutamate, and GABA [11]. Specifically, the serotonergic system has been considered to be the main target of antidepressant drugs such as selective serotonin reuptake inhibitors (SSRIs). Nevertheless, despite their clinical efficacy, these agents are often associated with several undesirable effects such as insomnia, nausea, diarrhea, anorexia and dry mouth [12], which may limit therapeutic effectiveness.

Consequently, there has been growing interest in alternative treatment strategies, particularly the use of natural products as potential antidepressant agents, including several such compounds, such as resveratrol [13] and curcumin [14]. In particular, gamma-oryzanol (GO), derived from rice bran oil, comprises cycloartenyl ferulate, 24–methylene cycloartanyl ferulate (MCAF) and campesteryl ferulate [15], which are reported to be safe for consumption with no serious side effects [16,17]. Beyond its safety profile, GO has several pharmacological properties in animal models, including anxiolytic-like effects accompanied by increased monoamine levels [18,19], as well as antidepressant-like effects [20,21]. However, the underlying mechanism of these pharmacological properties, especially its antidepressant effects, remains to be fully elucidated.

GO is highly lipophilic and exhibits poor water solubility, which limits its absorption, distribution, and bioavailability in biological systems, ultimately reducing its effectiveness as a pharmacological agent [22,23]. As a consequence, relatively high doses are often required to achieve therapeutic efficacy. To overcome this pharmaceutical limitation, GO solid dispersion (GOSD) has been successfully developed with substantially enhanced water solubility and improved dissolution physicochemical characteristics. Our previous in vitro findings support this approach, showing that a GO solid dispersion formulation improved water solubility to 1.37 ± 0.05 mg/mL. Furthermore, in Dx-treated SK-N-SH cells, GOSD showed neuroprotective effects at substantially lower GO-equivalent concentrations than free GO, with free GO requiring approximately 10–100-fold higher concentrations to achieve comparable effects [24]. However, the effects of GOSD in an in vivo Dx-induced depressive-like model and the molecular changes associated with its effects remain unclear. Therefore, this study investigated the antidepressant-like effects of GOSD in Dx-induced depressive-like behaviors in rats and further explored the associated molecular changes using proteomic analysis.

## 2. Results

### 2.1. Effects of GOSD on Body Weight

Body weight did not differ significantly among the control groups (Control, Control-GOSD5, and Control-GOSD10) under baseline conditions across all time points. Analysis revealed no significant treatment × time interaction [F (4.469, 24.582) = 0.376, *p* = 0.842].

For therapeutic evaluation, two-way repeated-measures ANOVA revealed a significant treatment × time interaction among the groups [F (8.607, 43.035) = 37.940, *p* < 0.001]. Subsequent Bonferroni-adjusted post hoc analysis revealed that while baseline measurements at day 1 and day 7 were comparable across all groups, Dx administration produced a marked decline by day 21 and day 28 compared with the Control group (all *p* < 0.001). However, administration of GOSD (5 and 10 mg/kg) or fluoxetine (Flu) did not significantly reverse or alter these values compared with the Dx group at any time point (Table 1).

### 2.2. Effects of GOSD on Depressive-like Behaviors in Rats

#### 2.2.1. Open Field Test (OFT)

Locomotor activity observed in the OFT showed no significant differences. Other behavioral measures, including total travel distance, number of crossings, and time spent in the central and peripheral zones, were comparable across experimental groups. All OFT data are provided in Table S1.

#### 2.2.2. Forced Swim Test (FST)

FST performance-comprising immobility time [F (2, 11) = 1.64, *p* = 0.24], latency to immobility [F (2, 11) = 0.78, *p* = 0.48], and immobility frequency [F (2, 11) = 1.11, *p* = 0.36] did not differ significantly among Control, Control-GOSD5, and Control-GOSD10 groups.

One-way ANOVA revealed an overall effect of treatment on immobility time under Dx-induced conditions [F (4, 20) = 11.99, *p* < 0.001]. Subsequent post hoc comparisons indicated longer immobility in the Dx group than in the Control group, whereas GOSD5, GOSD10, and Flu treatment significantly shortened immobility relative to the Dx group (Table 2).

The Dx group exhibited a significant reduction in the latency to the first immobility (31.80 ± 6.22 s) compared to the Control group (147.60 ± 11.99 s). The GOSD5-treated group exhibited a longer latency to the first immobility compared with the Dx group, an effect that was similar to the GOSD10-treated groups [F (4, 20) = 6.02, *p* = 0.002] (Table 2).

A significant group effect was observed for immobility frequency [F (4, 20) = 11.92, *p* < 0.001]. Immobility frequency was higher in the Dx group than in controls but was significantly lower following GOSD5, GOSD10, or Flu treatment compared with Dx alone (Table 2).

#### 2.2.3. Sucrose Preference Test (SPT)

Under baseline conditions, sucrose preference was not significantly different among the control groups throughout the experimental period. There was no significant treatment × time interaction [F (6, 30) = 0.183, *p* = 0.979].

**Figure 1:**
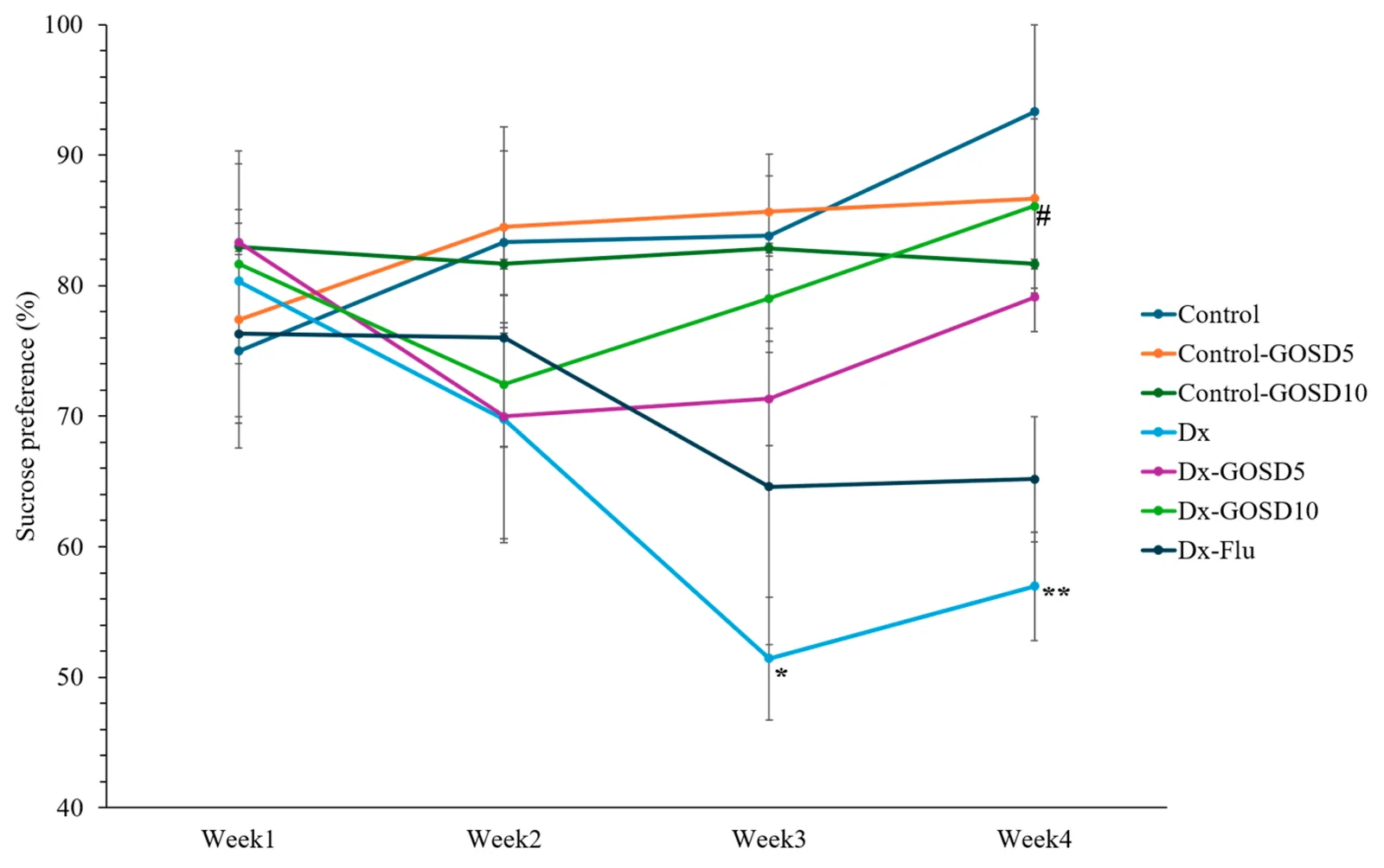
The effect of GOSD on SPT. Values are expressed as mean ± SEM. Statistical analysis was performed using two-way repeated-measures ANOVA, with treatment as the between-subjects factor and time as the within-subjects factor, separately for baseline and Dx-induced conditions. Bonferroni-adjusted pairwise comparisons were performed at each time point. (* *p* < 0.01, ** *p* < 0.001 when compared with the Control group, # *p* < 0.05, when compared with the Dx group).

Under Dx-induced conditions, a significant overall treatment effect on SPT was observed [F (4, 20) = 3.366, *p* = 0.029]. However, neither the main effect of time [F (3, 60) = 1.832, *p* = 0.151] nor the treatment × time interaction [F (12, 60) = 1.732, *p* = 0.082] reached statistical significance. In secondary Bonferroni-adjusted comparisons at individual time points, no significant group differences were detected on Days 7 or 14. The Dx group showed significantly lower sucrose preference than the Control group on Days 21 (*p* = 0.024) and 28 (*p* = 0.002). At Day 28, sucrose preference was significantly higher in the Dx-GOSD10 group than in the Dx group (*p* = 0.020), whereas Dx-GOSD5 and Dx-Flu did not differ significantly from the Dx group (Figure 1).

### 2.3. Effects of GOSD on Corticosterone Hormone Level

**Figure 2:**
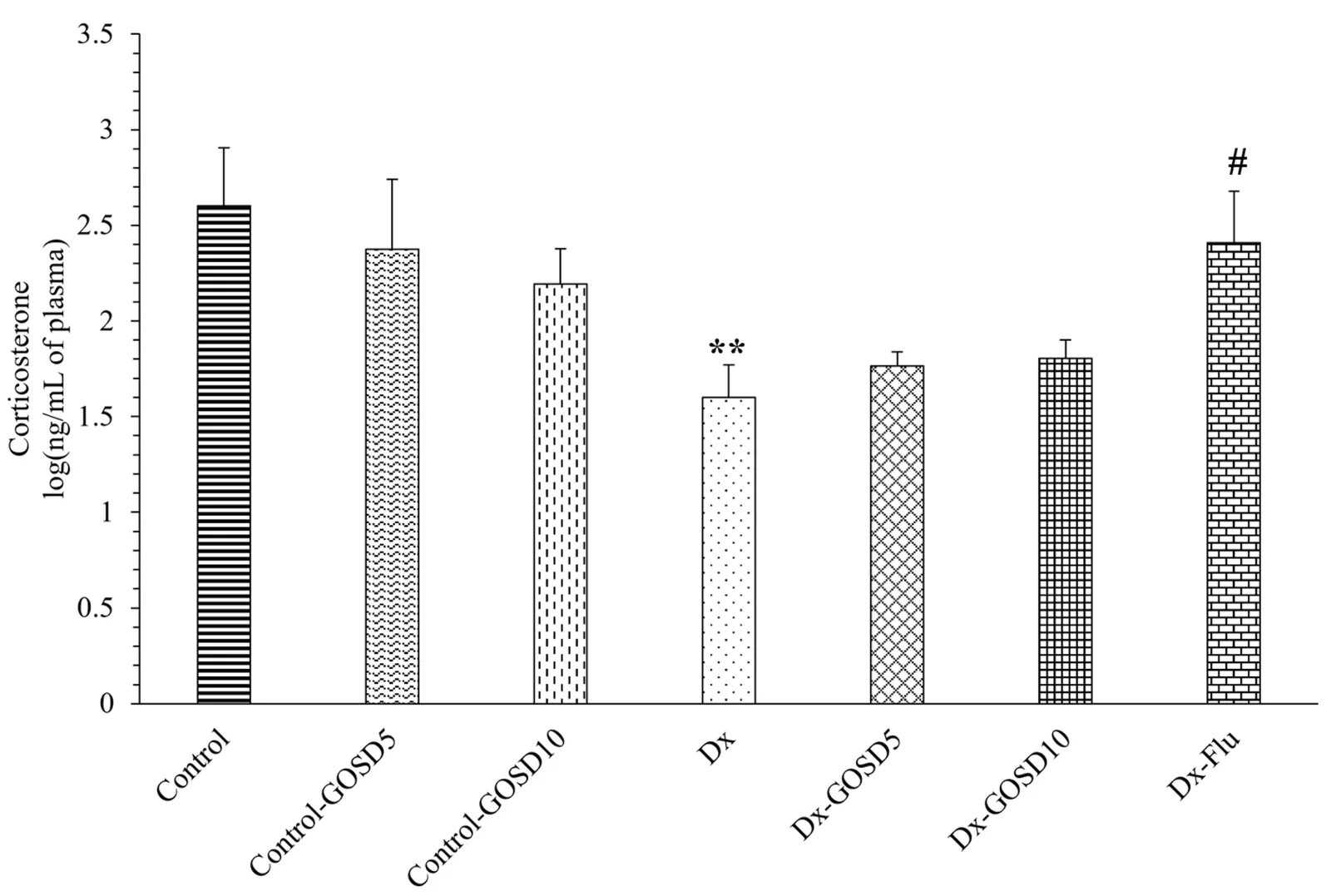
The effect of GOSD on plasma corticosterone levels. Values are expressed as mean ± SEM. Statistical analysis was performed by one-way ANOVA followed by Dunnett’s post hoc (** *p* < 0.01 when compared with the Control group, # *p* < 0.05 when compared with the Dx group).

Plasma corticosterone level did not differ significantly among the Control, Control-GOSD5, and Control-GOSD10 groups. Following Dx administration, plasma corticosterone levels were significantly reduced compared with the Control group [F (4,20) = 4.34, *p* = 0.011]. However, administration of GOSD at either 5 or 10 mg/kg did not produce significant changes relative to the Dx group. Conversely, Flu treatment significantly increased plasma corticosterone levels compared to the Dx group (Figure 2).

### 2.4. Effects of GOSD on Protein Profiles of the Frontal Cortex

#### 2.4.1. Identification and Quantification of Differentially Expressed Proteins (DEPs)

**Figure 3:**
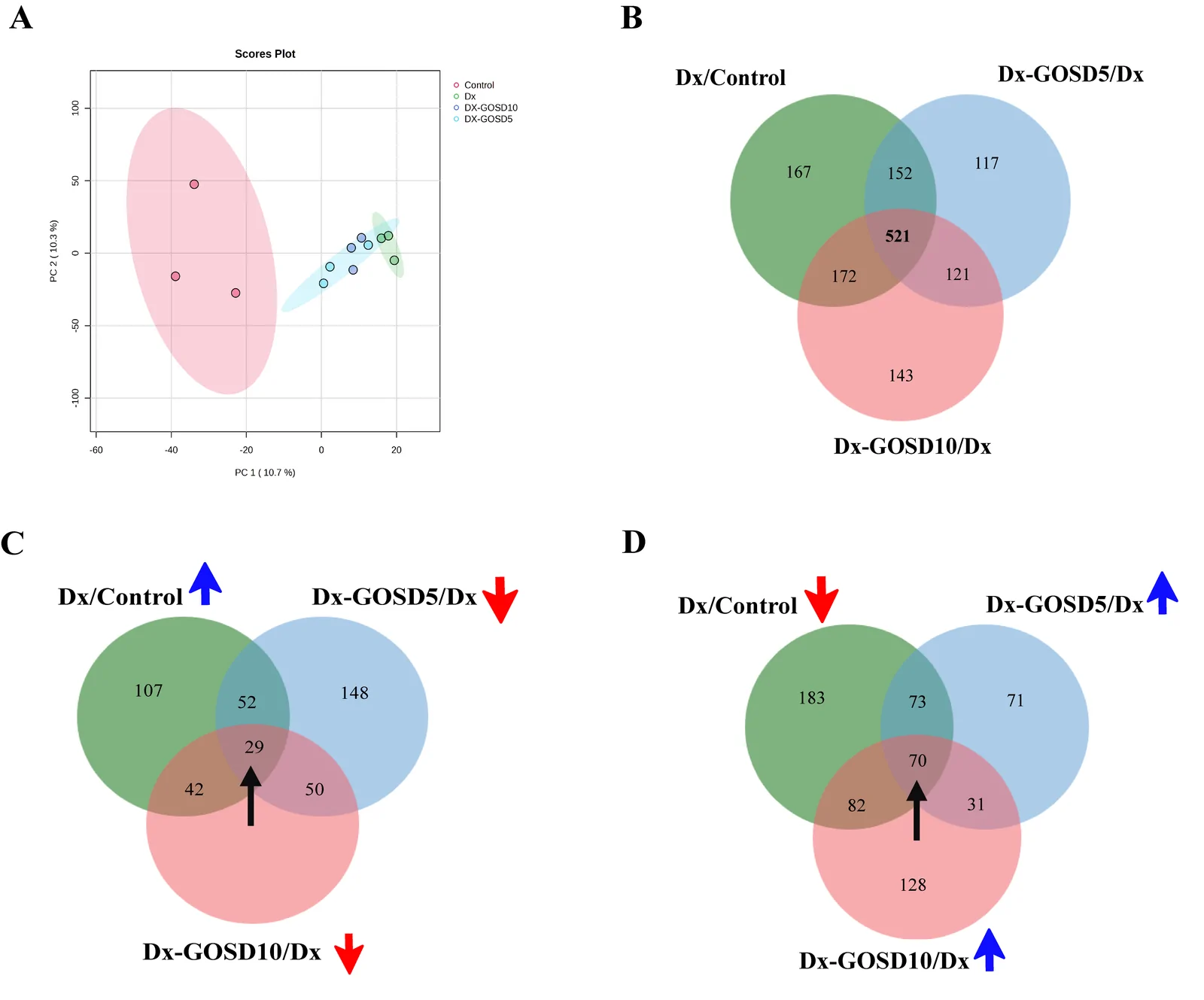
Principal component analysis (PCA) score plot displaying global proteomic distributions across samples (**A**). (**B**–**D**) Comparison of protein abundance patterns among Dx exposure and GOSD treatments. Venn diagram showing proteins commonly evaluated in the three pairwise comparisons: Dx/Control, Dx-GOSD5/Dx, and Dx-GOSD10/Dx. A total of 521 proteins were common to all three comparisons (**B**). Venn diagram illustrating the overlap of DEPs among the Dx/Control, Dx-GOSD5/Dx, and Dx-GOSD10/Dx groups (**C**). The black arrow shows overlapping proteins upregulated in the Dx/Control and downregulated in Dx-GOSD5/Dx and Dx-GOSD10/Dx (**D**). The black arrow indicates overlapping protein patterns of downregulated in Dx/Control and upregulated in Dx-GOSD5/Dx and Dx-GOSD10/Dx. Upregulated proteins are represented by blue arrows, while downregulated proteins are represented by red arrows.

Principal component analysis (PCA) was performed to visualize the overall distribution of the proteomic profiles. The first two principal components explained 10.7% and 10.3% of the total variance, respectively. The control samples showed a distinct distribution along PC1 compared with the Dx-treated conditions, whereas considerable partial overlap was observed among the Dx, Dx-GOSD5, and Dx-GOSD10 conditions (Figure 3A). Proteomic analysis of the frontal cortex identified 1883, 1707, 1632, and 1686 proteins in the Control, Dx, Dx-GOSD5, and Dx-GOSD10 groups, respectively, based on the predefined protein-identification criteria. Because the number of identified proteins differed among the experimental groups, pairwise comparisons were performed using only proteins that were commonly detected in both groups being compared. This approach allowed changes in protein abundance to be evaluated using quantitative values available for the same protein in both groups. Accordingly, 1012 proteins were included in the Dx/Control comparison, 911 proteins in the Dx-GOSD5/Dx comparison, and 957 proteins in the Dx-GOSD10/Dx comparison.

For each pairwise comparison, changes in protein abundance were determined from the abundance ratio between the two groups and expressed as a log_2_ fold change (log_2_FC). The comparison was calculated as log_2_(Dx/Control), log_2_(Dx-GOSD5/Dx), and log_2_(Dx-GOSD10/Dx), respectively. Therefore, a positiv_2_ log_2_FC indicates an increase in protein abundance, whereas a negati_2_e log_2_FC indicates a decrease in protein abundance relative to the comparison group. A predefined cutoff of log_2_FC > 1.2 or < −1.2 was used to classify proteins as DEPs. This fold-change criterion was used to identify proteins showing marked changes in abundance for further comparison across the experimental conditions. To identify proteins that could be followed across Dx exposure and both GOSD treatments, the protein lists from the three pairwise comparisons were compared using a Venn diagram. This analysis identified 521 proteins that were common to all three comparisons, indicating that these proteins could be evaluated across the Dx/Control, Dx-GOSD5/Dx, and Dx-GOSD10/Dx comparisons (Figure 3B). Among the 521 common proteins, 29 proteins showed increased abundance in the Dx/Control comparison but decreased abundance in both the Dx-GOSD5/Dx and Dx-GOSD10/Dx comparisons (Figure 3C, Table S2). This pattern indicates that these proteins were increased following Dx exposure but showed an opposite direction of change following GOSD treatment. Conversely, 70 proteins showed decreased abundance in the Dx/Control comparison but increased abundance in both GOSD-treated groups relative to the Dx group (Figure 3D, Table S3). Thus, these 70 proteins also showed an opposite abundance pattern between Dx exposure and GOSD treatment. Taken together, 99 proteins (29 + 70) showed opposite abundance patterns between the Dx/Control comparison and both GOSD/Dx comparisons. These 99 proteins were therefore selected for subsequent functional enrichment and pathway analyses to explore the biological processes and pathways associated with the protein abundance changes observed following GOSD treatment.

#### 2.4.2. Functional Annotation and Enrichment Analysis of DEPs

**Figure 4:**
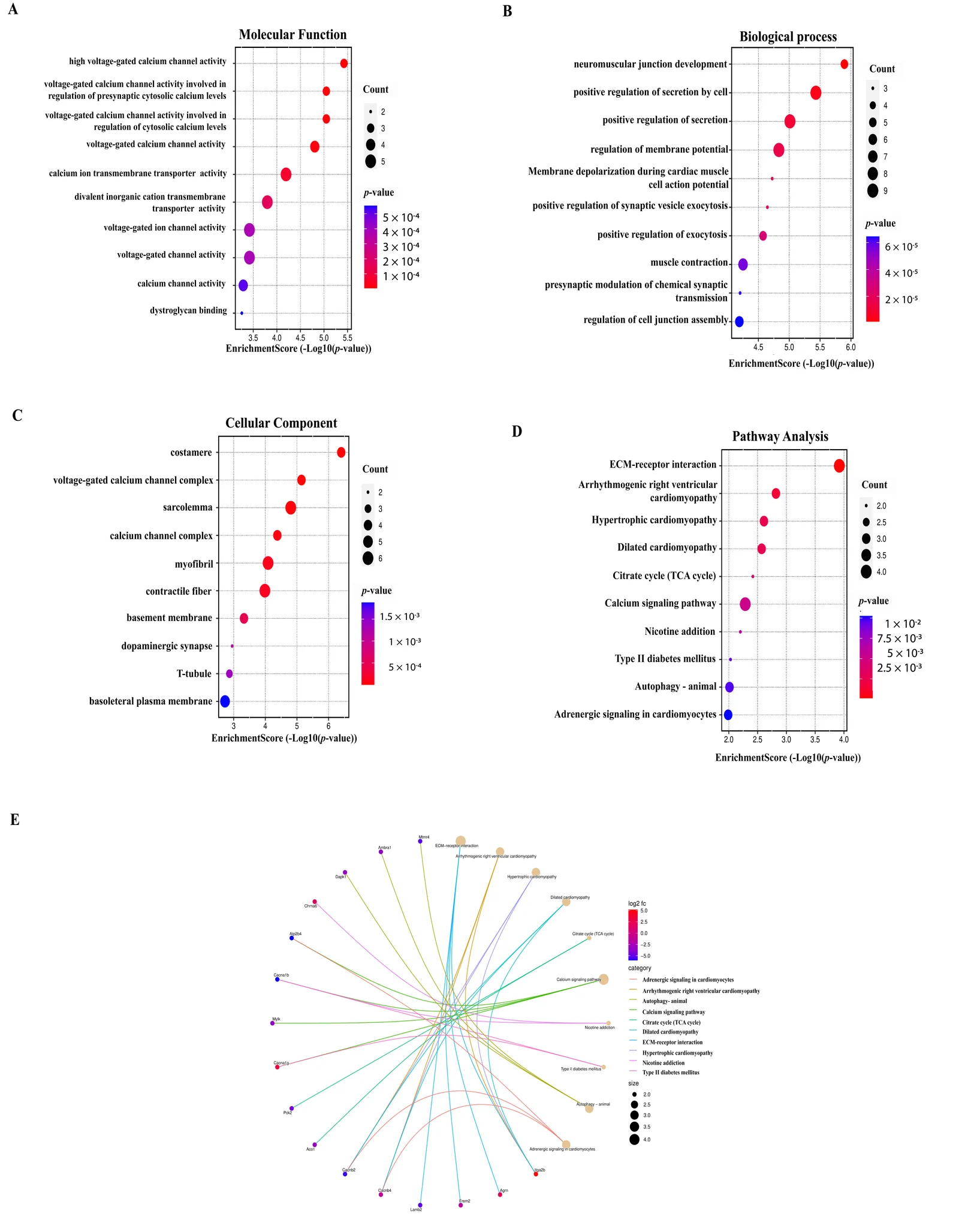
Overall proteomic profile and functional annotation of differentially expressed proteins (DEPs) in the frontal cortex. Gene Ontology (GO) enrichment analysis categorizing DEPs by Molecular Function (**A**), Biological Process (**B**), and Cellular Component (**C**). (**E**) Top 10 KEGG pathways identified via SRplot bioinformatic analysis. Enrichment scores are expressed as $-\log_{10}$ (*p*-value), dot sizes correspond to the number of proteins (Count) assigned to each term, and color intensity represents the enrichment *p*-value, with darker red indicating lower *p*-value (**D**). Cnet plot displaying protein–pathway linkages, where nodes represent individual proteins and edges denote pathway associations. Node color scale corresponds to $-\log_{10}$ (*p*-value), and node size scales with the number of associated genes (**E**).

To further investigate the biological alteration associated with Dx-induced depressive-like behaviors and the therapeutic effects of GOSD, a total of 99 DEPs were analyzed to characterize their functional associations. Gene Ontology analysis was performed across three main categories. In the molecular function (MF) category, DEPs were mainly enriched in voltage-gated calcium channel activity and transmembrane transporter activity (Figure 4A). For biological processes (BP), the most enriched terms included neuromuscular junction development, secretion, and regulation of synaptic transmission (Figure 4B). Within the cellular component (CC) category, the DEPs were primarily associated with the calcium channel complex and sarcolemma (Figure 4C). Additionally, KEGG pathway analysis using the SRplot bioinformatics tool revealed an enrichment in the calcium signaling pathway (Figure 4D). Four key proteins were identified, including Mylk (myosin light chain kinase, smooth muscle), Cacna1b (voltage-dependent N-type calcium channel subunit alpha), Atp2b4 (plasma membrane calcium-transporting ATPase 4), and Cacna1g (voltage-dependent T-type calcium channel subunit alpha-1G). The relationships between these proteins and the enriched KEGG pathways were further visualized using a Cnet plot, highlighting the calcium signaling pathway as a crucial mechanism potentially involved in the pathophysiology of depression and the action of GOSD (Figure 4E).

#### 2.4.3. The Protein–Protein Interaction (PPI) Network Construction and Visualization

**Figure 5:**
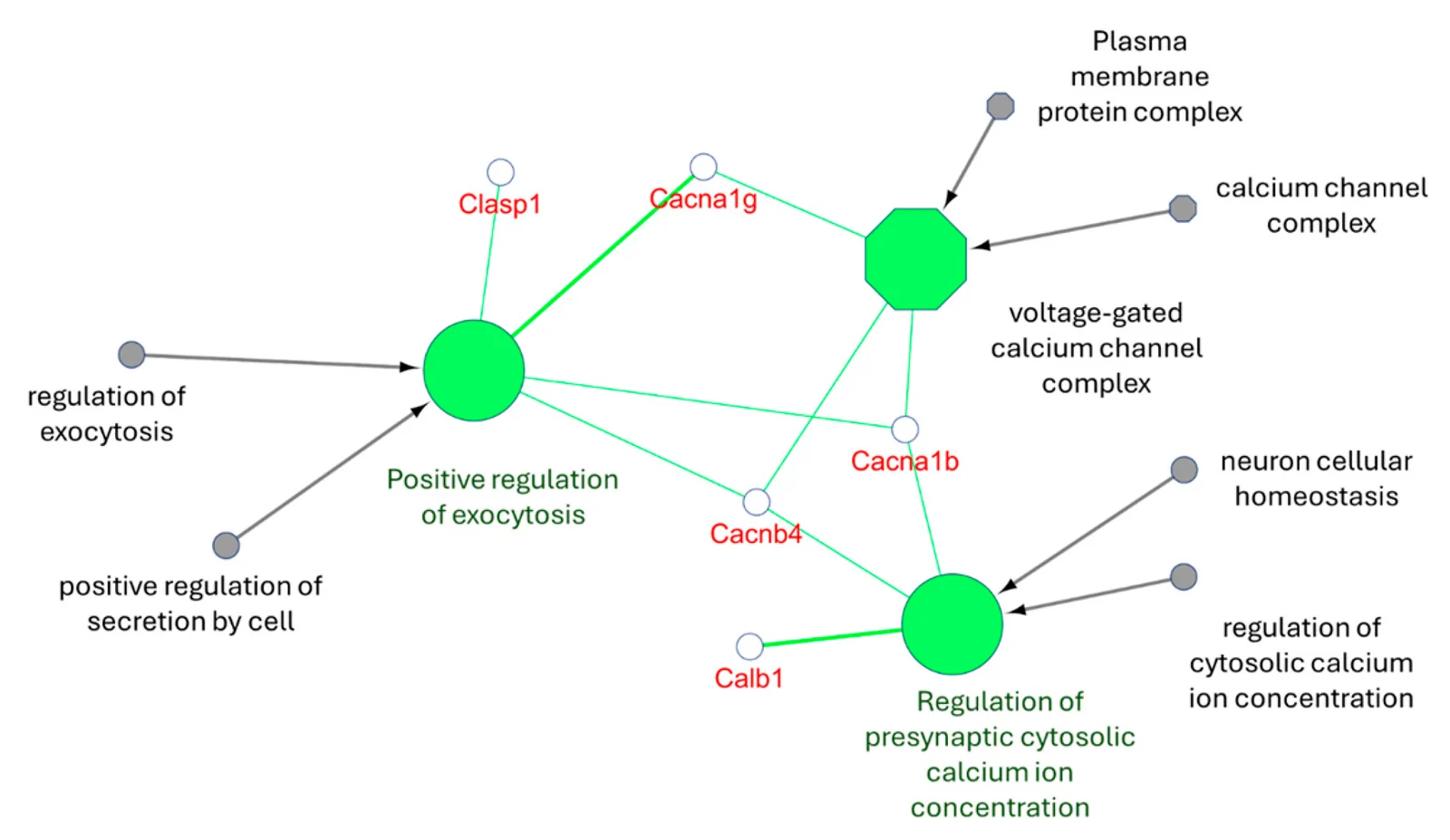
The DEPs were predicted to have PPI with CBP, such as Calb1, based on the PPI network data exported from the STRING database. Green lines link proteins to functional terms, while grey arrows link related terms.

Based on the results, a key KEGG pathway altered by GOSD treatment was further analyzed using Cytoscape (version 3.10.3) software with the ClueGO plugin to visualize the functional interconnections. The network was constructed to highlight interactions between four DEPs involved in the calcium signaling pathway and the calcium-binding protein (CBP), calbindin (Calb1). This analysis revealed the most prominent cluster within the PPI network, which was enriched for Gene Ontology terms such as positive regulation of exocytosis and regulation of presynaptic cytosolic calcium ion concentration, represented by green circles. In addition, KEGG pathway analysis revealed enrichment of proteins associated with the voltage-gated calcium channel complex, including Cacnb4, Cacna1g, and Cacna1b, represented by green hexagons. Notably, Cacnb4 and Cacna1b were closely associated with Calb1, suggesting a functional relationship within the calcium signaling cascade (Figure 5).

#### 2.4.4. Validation of Calcium-Binding Protein Calb1 Expression

**Figure 6:**
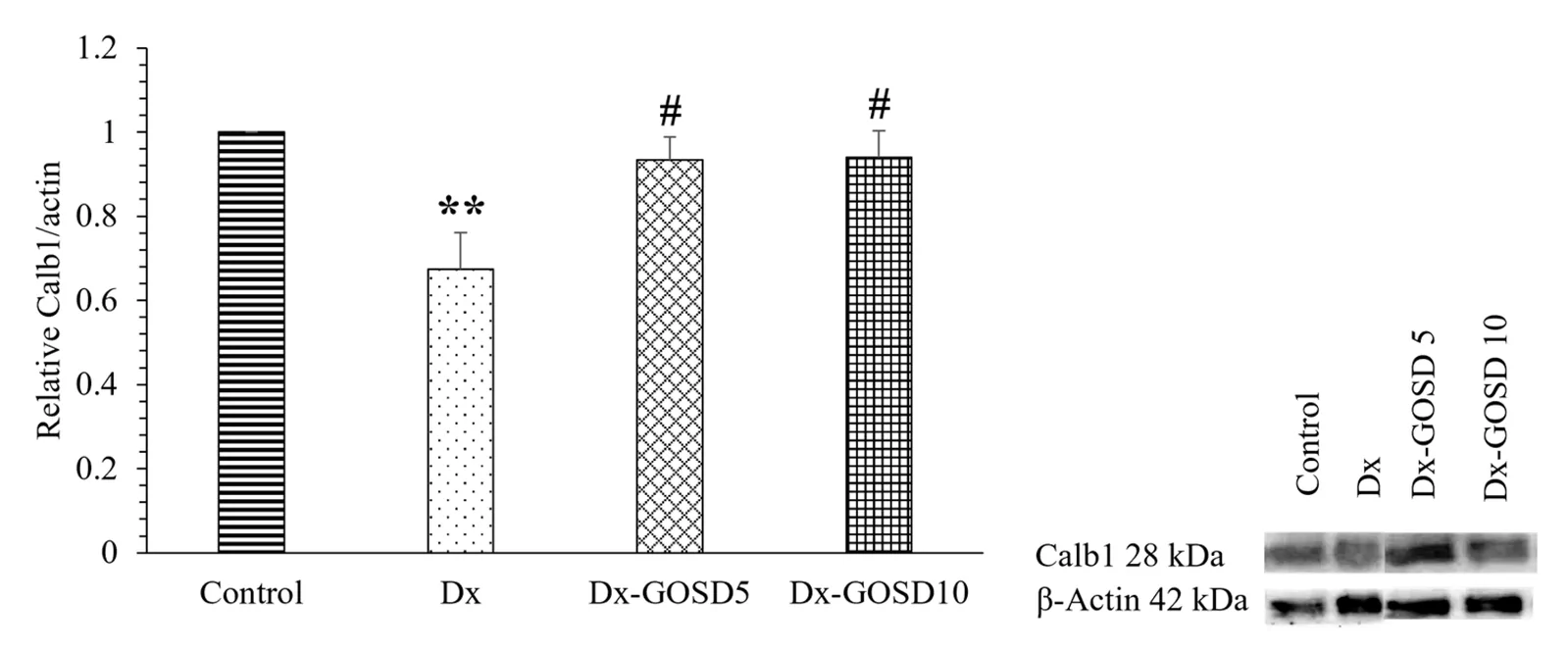
Quantitative analysis of Calb1 expression in rat frontal cortex (*n* = 5 animals per group). Representative Western blot images of Calb1 and beta-actin are shown. Calb1 band intensity was normalized to the corresponding beta-actin signal within the same lane and subsequently expressed relative to the corresponding control sample analyzed on the same membrane. The control was therefore assigned a fixed reference value of 1.0. Values are expressed as mean ± SEM. ** *p* < 0.01 vs. the normalized control reference (one-sample *t*-test); # *p* < 0.05 vs. the Dx group (one-way ANOVA followed by Dunnett’s multiple-comparison test). The blots shown were cropped from different parts of the same gel. Full-length blots are provided in Supplementary Figure S1.

As described in Section 2.4.3, the calcium signaling cascade was strongly implicated, and the calcium-binding protein Calb1, a protein marker of GABAergic neurons, was identified within the PPI network. Regarding the hypothesis that corticosterone-induced depression involves neurotransmitter imbalances, Calb1 was selected for further validation. Calb1 immunoreactivity was assessed using Western blotting. The results showed a significant decrease in the Dx group compared to the control [F (3,16) = 5.722, *p* = 0.007]. However, GOSD5 mg/kg and GOSD10 mg/kg treatments significantly increased Calb1 expression compared to the Dx group (Figure 6).

## 3. Discussion

The present study revealed that GOSD did not produce significant alterations in baseline body weight, behavioral, or endocrine parameters evaluated in the control animals. These findings suggest an absence of obvious adverse effects within the limited behavioral and endocrine markers assessed in this study. Chronic Dx administration induced pronounced depressive-like behaviors in rats, characterized by increased immobility time and frequency, reduced latency to first immobility in the FST, and decreased sucrose consumption in the SPT. These outcomes align with a previous report demonstrating that a stress-induced model displays reduced immobility latency and prolonged despair relative to controls [25]. The FST is a standardized assay for evaluating potential protective compounds, wherein immobility in an inescapable aquatic environment reflects a loss of escape motivation and behavioral despair [26], while the latency to initial immobility serves as an indicator of the animal’s primary coping effort against acute stress [27]. Notably, administration of GOSD (5 and 10 mg/kg) effectively attenuated these behavioral deficits by extending immobility latency, shortening immobility duration, and counteracting Dx-induced anhedonia, a core hallmark of depressive-like states characterized by diminished sucrose intake [9,10,28]. Taken together, these findings demonstrate the protective efficacy of GOSD against glucocorticoid-induced behavioral despair and anhedonia. In contrast, GOSD and fluoxetine did not improve SPT parameters under the present experimental conditions. Although fluoxetine is commonly used as a positive control in rodent models of depressive-like behaviors, its effects on sucrose preference are not uniformly observed across experimental paradigms. Previous studies in rats have similarly reported no significant restoration of sucrose preference following fluoxetine treatment; for example, no significant improvement in sucrose preference following fluoxetine treatment at 10 mg/kg [29]; chronic fluoxetine treatment improved other depression-related measures, including forced swim test immobility or corticosterone levels, without fully restoring sucrose preference [30,31]. Nevertheless, the lack of a significant fluoxetine response in the SPT limits the interpretation of the sucrose-preference findings and should be considered when interpreting the effects of GOSD on this behavioural endpoint. The results from locomotor activity did not significantly differ between the control group and the Dx group. This indicates that the observed antidepressant-like effects of GOSD in the FST and SPT were not due to changes in overall motor function. These results are consistent with a prior study showing that Dx-induced depressive-like behavior models often affect mood-related behaviors without significantly impairing locomotor activity [9]. In addition to behavioral alteration, repeated Dx administration induced a significant reduction in body weight, consistent with previous findings that glucocorticoid treatment led to body weight loss during the first week of the treatment phase, reflecting substantial systemic effects [32]. However, GOSD treatment did not affect body weight, suggesting that its protective behavioral effects were independent of metabolic alterations. Furthermore, locomotor activity in OFT did not differ significantly between the Control and Dx groups, suggesting that the elevated immobility duration in the FST was not merely an artefact of impaired motor function. Nevertheless, other systemic influences of prolonged Dx exposure cannot be fully ruled out. Parallel to these outcomes, plasma corticosterone levels were significantly suppressed in the Dx-treated group compared to controls.

Dx is a potent synthetic glucocorticoid receptor agonist that can suppress endogenous HPA-axis activity through negative feedback. Consistent with this effect, a previous study in rats has demonstrated suppression of circulating ACTH and corticosterone following repeated Dx administration [33]. Importantly, reduced endogenous corticosterone does not necessarily indicate reduced overall glucocorticoid signaling, as exogenous Dx continues to activate glucocorticoid receptors. Previous studies have reported that repeated Dx exposure can induce depressive-like behaviors while also reducing circulating corticosterone [9,10]. Therefore, the reduction in endogenous corticosterone and the depressive-like behavioral alterations observed in this model may represent associated consequences of repeated dexamethasone exposure, and the present findings do not establish a causal relationship between reduced corticosterone and the depressive behavioral phenotype. Although alterations in corticosterone homeostasis may influence neurotransmitter regulation and neuronal function [34], the extent to which reduced corticosterone contributes to the behavioral alterations observed in this Dx model remains unclear. Interestingly, fluoxetine treatment significantly increased corticosterone levels compared with the Dx group, suggesting a partial attenuation of Dx-induced suppression of endogenous corticosterone. This finding is consistent with a previous report showing that SSRIs can influence HPA-axis activity and glucocorticoid regulation [35]. In contrast, GOSD did not significantly alter plasma corticosterone levels compared with the Dx group. Thus, the behavioral effects observed following GOSD treatment were not accompanied by normalization of circulating corticosterone under the present experimental conditions. However, because other components of HPA-axis regulation, including corticotropin-releasing hormone, adrenocorticotropic hormone, glucocorticoid receptor-related signaling, and feedback sensitivity, were not assessed, the involvement of HPA-axis modulation in the effects of GOSD remains unclear. Previous studies have supported that GO exhibits antidepressant-like effects through modulation of monoaminergic neurotransmission [18] and nitric oxide synthase signaling [21], both of which are involved in neuronal function and plasticity. Nevertheless, the molecular changes associated with the behavioral effects of GOSD remain incompletely understood. To further explore these changes, an exploratory proteomic analysis was performed in the Dx, Dx-GOSD5, and Dx-GOSD10 groups. The proteomic findings identified changes in proteins associated with several biological pathways, including calcium signaling. These findings suggest a potential association between GOSD treatment and calcium-signaling-related molecular changes. The DEPs showed changes across the Dx/Control, Dx-GOSD5/Dx, and Dx-GOSD10/Dx comparisons. Among these DEPs, Gene Ontology analysis highlighted associations with the calcium signaling pathway, which is critical for neuronal excitability, synaptic plasticity, and neurotransmitter release [36]. Four proteins, Mylk, Cacna1b, Atp2b4, and Cacna1g were identified as components of this pathway, with ClueGO network analysis further linking these proteins to Calb1, a CBP implicated in GABAergic neurotransmission. KEGG pathway enrichment analysis identified the calcium signaling pathway as being associated with the protein abundance changes observed following Dx and GOSD treatments, including changes involving Calb1. The reduced Calb1 expression observed in the Dx group may reflect molecular alterations associated with GABAergic neuronal function [37,38]. As a GABA marker and critical regulator of calcium buffering, Calb1 plays an essential role in maintaining synaptic stability in inhibitory interneurons [38]. Consistent with previous studies, reduced Calb1 expression has been reported in animal models of depression and post-mortem brains of individuals with mood disorders, supporting its role in depression-related neurobiological alterations [37,38]. Notably, reduced Calb1 expression in the orbitofrontal cortex has been linked to functional and morphological impairments of GABAergic neurons in depression [37,39]. This evidence aligns with broader findings indicating that dysfunction of CBP contributes to abnormal calcium signaling, impaired synaptic communication, and heightened vulnerability to depression-related neuronal damage [40,41]. The observed increase in Calb1 immunoreactivity following GOSD 5 mg/kg and GOSD 10 mg/kg treatments suggests that the effects of GOSD may be associated with alterations in calcium- and GABA-related neuronal processes. Given that the CBP of Calb1 contribute to neuronal resilience against excitotoxicity [42], its upregulation may be associated with the antidepressant-like effects of GOSD.

Calcium homeostasis is primarily maintained through voltage-gated calcium channels (VGCCs), which are essential for neuronal excitability, neurotransmitter release, and gene expression, making them potential therapeutic targets for psychiatric disorders [43,44,45]. These channels regulate calcium influx into neurons in response to changes in membrane potential. Notably, Cav2.2 channels (encoded by Cacna1b) are located presynaptically, where they control calcium entry and trigger GABA release [46,47]. Dysregulation of VGCCs has been implicated in neuropsychiatric conditions such as schizophrenia by reducing the Cacna1g gene level [48]. Myosin light chain kinase (MLCK) modulates intracellular calcium levels due to neuronal activity, binding to calcium/calmodulin (CaM) signaling [49]. Plasma membrane calcium ATPase (PMCA) is an ATPase enzyme that pumps calcium ions to help maintain calcium homeostasis between the intracellular and extracellular environments [50]. This enzyme is essential in maintaining the equilibrium of Ca^2+^ ions, which are vital for the establishment and preservation of synaptic connections [51]. A previous study found that PMCAs were expressed in parvalbumin-positive GABAergic presynaptic terminals in the cortex [52]. Dysregulation of calcium signaling has been linked to GABAergic dysfunction, a well-established mechanism underlying depression [37,53]. Reduced GABAergic transmission is associated with depressive symptoms, making it a potential therapeutic target. The findings from this study suggest that GOSD treatment is associated with alterations in CBPs, including Calb1. These molecular changes may reflect the involvement of calcium-related pathways and may be associated with the observed attenuation of depressive-like behaviors.

Several limitations should be considered. First, the relatively small sample size (*n* = 4–5 animals per group) may limit the precision of the estimated effects and the ability to detect small-to-moderate differences between groups. Nevertheless, comparable sample sizes have been used in previous rodent studies assessing depression-related behavioral outcomes [54,55]. Second, the Dx solution contained 5% DMSO, whereas the control groups received saline alone. DMSO was used to dissolve Dx, and 5% DMSO has previously been used for subcutaneous Dx administration in adult rats [56,57]. The concentration used in this study was substantially lower than the 32% and 64% DMSO concentrations previously reported to significantly affect locomotor activity [58], suggesting that 5% DMSO was unlikely to induce substantial stimulatory effects on brain activity. Nevertheless, the effects of this concentration on central nervous system activity were not directly assessed, which represents a limitation of the present study. This limitation should therefore be considered when interpreting effects attributed to dexamethasone. Another limitation is the absence of free GO and blank-carrier control groups. However, GOSD and free GO have been compared in vitro in our previous study [24]. The present study extends these findings by providing in vivo evidence of the effects of GOSD on depression-like behaviors, thereby supporting the antidepressant efficacy of the GOSD formulation. In addition, biological samples were pooled for LC–MS/MS proteomic analysis; therefore, statistical differential-abundance analysis based on independent biological replicates could not be performed. Protein selection was instead based on fold-change criteria, as used in previous exploratory proteomic studies with pooled samples [59,60]. Accordingly, the proteomic findings are interpreted as exploratory and provide a basis for further investigation using independent biological samples. Finally, although changes in Calb1 expression were confirmed by Western blot analysis, intracellular calcium dynamics and GABAergic function were not directly assessed, and the functional role of Calb1 was not investigated. Therefore, the present findings establish molecular associations rather than a causal role of Calb1 or calcium signaling in the behavioral effects of GOSD.

In conclusion, the present study showed that GOSD attenuated dexamethasone-induced depressive-like behaviors in rats, as demonstrated by improvements in the forced swim and sucrose preference tests. Proteomic analysis identified alterations in several proteins associated with calcium-related processes following GOSD treatment. Among these proteins, Calb1 was identified as one of the altered molecular targets. These findings suggest an association between GOSD treatment and calcium-related molecular alterations, including changes in Calb1 expression. Overall, these findings support further investigation of GOSD as a potential complementary approach for dexamethasone-induced depressive-like behaviors.

## 4. Materials and Methods

### 4.1. Preparation of Gamma-Oryzanol Solid Dispersion

GOSD was prepared according to the previously described method [24]. In brief, GO, polyvinyl pyrrolidone K30 (PVP K30), and lecithin were melted together with the aid of isopropyl alcohol. Then, the resulting mixture was immediately cooled down, and the solvent was removed using a hot air oven at 45 °C. After complete drying, the GOSD was ground into a fine powder. The dried GOSD appeared as a white powder containing GO of 12.77 ± 0.40%, with a high % GO recovery of ~90%. Upon dilution in water, GOSD exhibited an increase in water solubility of GO to 1.42 ± 0.12 mg/mL, with a mean particle size of 71.20 ± 3.90 nanometers.

### 4.2. Animals and Treatment

**Figure 7:**
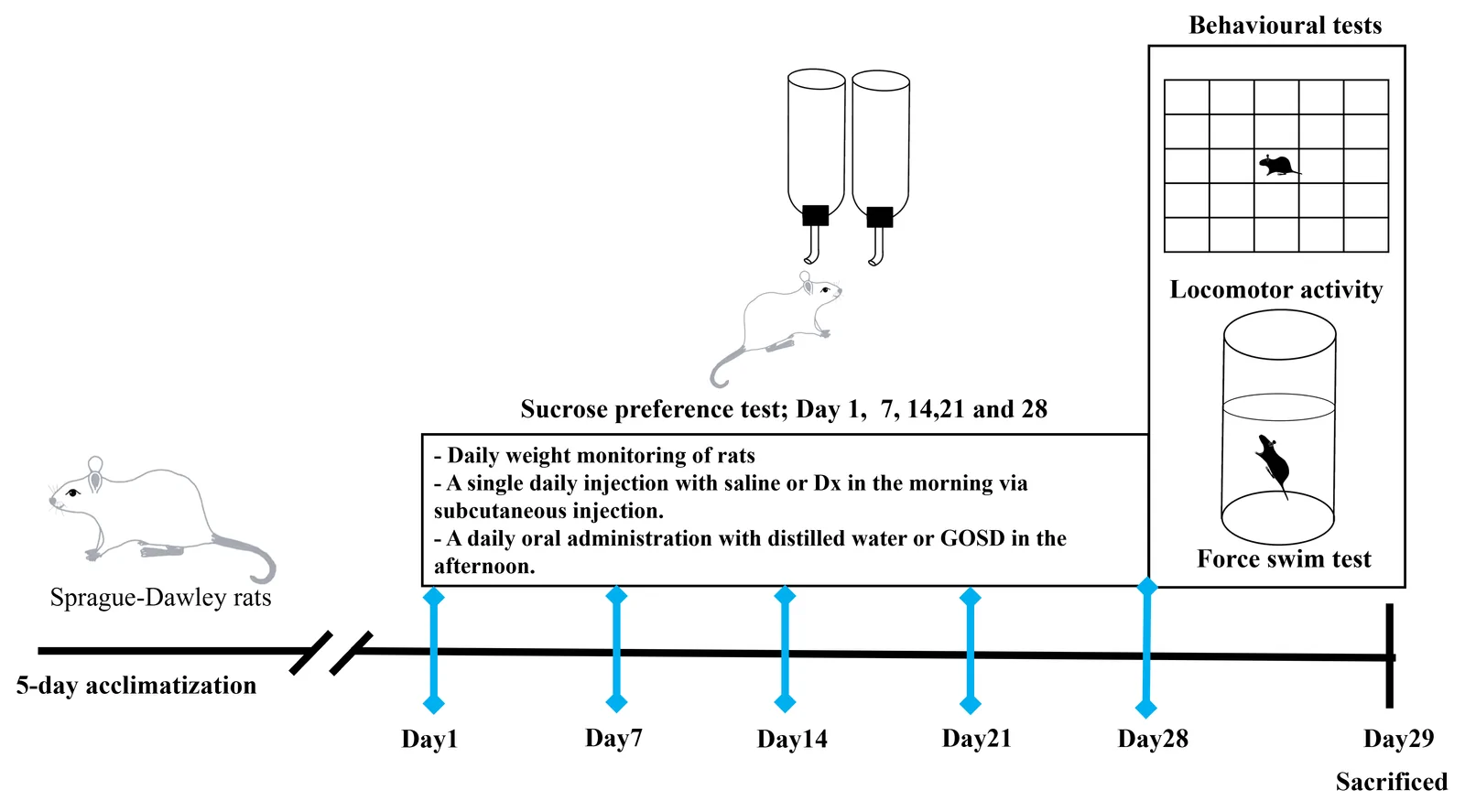
Diagrammatic overview of experimental schedule and protocol.

Six-week-old male Sprague-Dawley rats 180–220 g were obtained from Nomura Siam International Co., Ltd. (Bangkok, Thailand). Before the experiment, rats were allowed five days to adapt to the new environment. Rats were kept in groups of four per cage under controlled environmental conditions (22 ± 1 °C, 55 ± 10% humidity, 12-h light/dark cycle) with ad libitum access to standard food and water. Experimental procedures were conducted in accordance with NUCAR guidelines and ARRIVE standards, under protocol approval from the Naresuan University Animal Care and Use Committee (No. NU-AE620513, 22 July 2018). Daily body weights were measured for all animals. Animals were divided into seven groups (*n* = 4–5): Control, Control-GOSD5, Control-GOSD10, Dx (1.5 mg/kg), Dx-GOSD5 (5 mg/kg GOSD), Dx-GOSD10 (10 mg/kg GOSD), and Dx-Flu (10 mg/kg fluoxetine). Reverse osmosis (RO) water served as the vehicle for the Control and Dx groups. Treatments lasted for 28 days, consisting of morning subcutaneous injections (Dx or saline) and afternoon oral gavages. For administration, GOSD and fluoxetine were freshly prepared in RO water. GOSD was administered at formulation doses of 5 and 10 mg/kg/day (equivalent to approximately 0.64 and 1.28 mg/kg/day of gamma-oryzanol) using final GOSD concentrations of 0.5 and 1.0 mg/mL, respectively. Following the manufacturer’s guidelines (APExBIO Technology, LLC, Houston, USA), Dx stock solution (15 mg/mL in DMSO) was diluted with sterile 0.9% saline to 0.75 mg/mL, resulting in 5% (*v/v*) DMSO, a concentration previously used for subcutaneous dexamethasone administration in rats [56,57] (Figure 7).

### 4.3. Dosage Information

GOSD dose selection was guided by our previous preclinical studies [24]. The selected doses of 5 mg/kg and 10 mg/kg body weight/day were converted to human equivalent dose (HED) using the standard Km factor (rat = 6; human = 37), as described previously [61]. This conversion yielded HEDs of approximately 0.81 mg/kg and 1.62 mg/kg, respectively. For a 60-kg adult, these values correspond to daily doses of 48.6 mg and 97.2 mg. Considering the 12.77% gamma-oryzanol content of the formulation, these values correspond to gamma-oryzanol-equivalent HEDs of approximately 0.10 and 0.21 mg/kg/day, or approximately 6.2 and 12.4 mg gamma-oryzanol/day for a 60-kg adult, respectively. The dosages of Dx were selected based on previous studies [9,62,63]. The Flu dosage was determined according to established protocols from earlier research [9,64,65].

### 4.4. Behavioral Tests

#### 4.4.1. OFT

Locomotor activity was evaluated in an open field test. The apparatus comprises an arena enclosed by 40 cm high walls to prevent escape. The floor of the open field is divided into squares. Each animal was positioned at the center of the open field, where the apparatus floor was 72 cm. Following that, a 10-min video was recorded, and behavior was observed for the last 5 min. Afterwards, the number of line crossings, total distance, and the percentage of time spent in each zone (center and peripheral) were analyzed using V Smart 3.0 software (SMART; Panlab Co., Barcelona, Spain).

#### 4.4.2. FST

The animals were placed in a transparent acrylic cylinder filled with tap water at a temperature range of 23–27 °C for 6 min (360 s), and the entire session was recorded on video. Immobility time was defined as the period during which the rat showed no movement or slight movements while keeping the nose above water and was tracked during the last 5-min observation period. Immobility time, latency time, and behavioral frequencies were analyzed. The duration measures the sum of time in each behavior pattern within a given period, whereas frequency is a count of behavior patterns. Immobility is defined as a rat remaining motionless, still floating in the water, performing only the essential movement to keep its head above the water. Swimming was recorded as an active movement: swimming or diving around the cylinder. Climbing was determined as active forepaw movement against the wall [66].

#### 4.4.3. SPT

This performance was tested for anhedonia testing behavior. The rat was single-housed and kept in proper cages with two bottles of 1% (*w/v*) sucrose solution for 24 h before the test. Then, the rat was housed individually in cages with 1 bottle of water and 1 bottle of 1% (*w/v*) sucrose solution with free access. The volumes of remaining sucrose and water were determined 1 h later, sucrose preference index (%) was measured. To minimize potential side preference, the positions of the sucrose and water bottles were randomly switched during the sucrose preference test.

### 4.5. Corticosterone Level

Following the completion of behavioral testing, animals were returned to their home cages. Blood and brain tissue samples were collected 24 h after FST. Blood was collected approximately 24 h after the FST. The interval between the final behavioral test and sample collection was standardized across all experimental groups. Blood was obtained by cardiac puncture and immediately transferred into heparin-coated tubes. The sample was centrifuged at 4000 *g* for 10 min at 4 °C. Then, supernatants were collected for plasma. The alteration of hormone level was assessed by using an enzyme-linked immunosorbent assay (ELISA) kit (AB108821; Abcam, Cambridge, UK) with a pre-coated polyclonal antibody specific for corticosterone onto a 96-well microplate. The absorbance of each sample was measured at a wavelength of 450 nm.

### 4.6. Proteomic Analysis

#### 4.6.1. Protein Extraction and Protein Digestion

On Day 29 of the experiment, brain tissues were collected 24 h after the FST. Frontal cortex tissues from the Control, Dx, Dx-GOSD5, and Dx-GOSD10 groups were pulverized in liquid nitrogen. For each sample, 100 mg of fine powder was homogenized in 450 µL of 0.5% SDS, vortexed for 1 h, and centrifuged at 10,000× *g* for 15 min. The supernatant was collected, mixed with 900 µL of cold acetone, and incubated overnight at −20 °C to precipitate proteins. Following incubation, the mixture was centrifuged at 10,000× *g* for 15 min; the supernatant was discarded, and the resulting protein pellet was air-dried and stored at −80 °C. Total protein concentration was quantified using the Lowry method with bovine serum albumin (BSA) as the standard [67]. Samples from each group were used in equal protein amounts and performed in-solution digestion. 5 µg of protein per sample was fully reconstituted in 10 mM ammonium bicarbonate (AMBIC). Disulfide bonds were reduced with 5 mM dithiothreitol (DTT) in 10 mM AMBIC at 60 °C for 1 h, followed by alkylation of sulfhydryl groups using 15 mM iodoacetamide (IAA) in 10 mM AMBIC for 45 min at room temperature in the dark. Proteins were digested with sequencing-grade porcine trypsin at a 1:20 (*w/w*) enzyme-to-protein ratio for 16 h at 37 °C. The resulting tryptic peptides were dried under vacuum and resuspended in 0.1% formic acid prior to nano-liquid chromatography-tandem mass spectrometry (nanoLC-MS/MS) analysis.

#### 4.6.2. Liquid Chromatography Tandem-Mass Spectrometry Analysis

Liquid chromatography-tandem mass spectrometry (LC-MS/MS) was performed to characterize the proteomic profiles of the control, Dx, and GOSD groups (*n* = 1 pooled biological replicate per group). To minimize technical variability, each peptide digest was injected in technical triplicate. For each biological group, the median protein intensity across the three technical replicates was calculated and used as the representative expression value without prior quality-control filtering across injections. Comparative quantitative and statistical analyses were subsequently conducted across the representative biological values. Tryptic peptide samples were analyzed using an Ultimate 3000 RSLCnano System coupled to a Hybrid Quadrupole-Time-of-Flight (Q-TOF) Impact II™ mass spectrometer equipped with a Nano-CaptiveSpray ion source (Bruker Daltonics, Breman, Germany). Briefly, 100 ng of peptide digest was trapped and desalted on a C18 PepMap 100 µ-precolumn (300 µm i.d. × 5 mm, 5 µm, 100 Å; Waltham, MA, USA, Thermo Scientific). Peptides were then separated on an Acclaim PepMap RSLC C18 analytical column (75 μm I.D. × 15 cm, 2 μm, 100 Å nanoViper; Waltham, MA, USA, Thermo Scientific) maintained at 60 °C. Mobile phase A consisted of 0.1% formic acid in water, and mobile phase B contained 0.1% formic acid in 80% acetonitrile. Elution was performed at a constant flow rate of 0.30 μL/min using a linear gradient of 5–55% solvent B over 30 min. Electrospray ionization was set to 1.6 kV via the CaptiveSpray source, with nitrogen utilized as a drying gas at a flow rate of 50 L/h. Tandem mass spectra were acquired via collision-induced dissociation (CID) using nitrogen as the collision gas. Peptide fragment mass spectra were acquired in data-dependent AutoMS mode with selection of the most abundant precursor ions in a 3-s cycle for fragmentation (Compass 1.9 acquisition and processing software, Bruker Daltonics, Breman, Germany). Electrospray ionization was performed at 1.6 kV using a CaptiveSpray source. Full MS and MS/MS spectra were acquired in positive-ion mode at 2 Hz over *m/z* 150–2200. Collision energy was linearly ramped between 23–65 eV as a function of precursor *m/z*, increasing from 23 eV at low *m/z* to 65 eV at high *m/z*, to ensure efficient and consistent fragmentation across the full mass range.

#### 4.6.3. Bioinformatic Analysis

Protein identification and label-free quantification (LFQ) were performed using MaxQuant software (version 2.5.0.0) equipped with the Andromeda search engine [68]. Spectra were searched against the *Rattus norvegicus* reference proteome database retrieved from UniProt. The search parameters were configured with trypsin specified as the cleavage enzyme, allowing up to two missed cleavages. Mass tolerances were set to 20 ppm for the initial precursor search and 4.5 ppm for the main search. Carbamidomethylation of cysteine was designated as a fixed modification, while methionine oxidation and N-terminal protein acetylation were set as variable modifications, with a maximum of five modifications allowed per peptide. False discovery rates (FDR) for both peptides and proteins were controlled at 1% using a target-decoy reversed database strategy. Protein identification required a minimum peptide length of seven amino acids and at least two identified peptides per protein, including at least one unique peptide.

Exploratory multivariate analysis and data visualization were performed using MetaboAnalyst version 6.0 [69]. PCA was performed to visualize the overall protein abundance patterns and assess the analytical reproducibility of the technical LC–MS/MS injections. Protein abundance data were processed in MetaboAnalyst using standard protocol. Non-informative features were removed using an interquartile range (IQR) filter (40%) and a low-abundance filter based on median intensity (10%). Zero values representing undetected signals were replaced with one-fifth of the minimum positive value for the corresponding protein. The data were subsequently normalized by median normalization and subjected to Pareto scaling (mean-centered and divided by the square root of the standard deviation).

Protein interaction network analysis and functional annotation of proteins altered following GOSD treatment were performed using the SRplot bioinformatics platform (https://www.bioinformatics.com.cn/, accessed on 24 April 2026). The top ten GO terms were rendered as a bar plot using the online SRplot tool. The platform is a web tool that is freely accessible and provides enrichment of Gene Ontology terms and Kyoto Encyclopedia of Genes and Genomes (KEGG) annotation [70]. Proteins with a log_2_ fold change (log_2_FC) > 1.2 or < −1.2 were classified as DEPs based on the predefined fold-change criterion and were used for subsequent bioinformatics analyses. This fold-change criterion has similarly been used for descriptive protein selections in exploratory proteomic studies [59,60].

The protein–protein interaction was analyzed by the STRING database (http://www.string-db.org/, accessed on 24 April 2026). The DEPs from the experiment were imported into the STRING website and sent into Cytoscape software (version 3.10.2) for generating and visualizing the protein interaction network for *Rattus norvegicus* species. The Cytoscape ClueGO plugin was used to find clusters in PPI networks involving Gene Ontology terms and KEGG pathways, and different shapes are automatically assigned to individual clusters to aid in visualizing the protein origins within the network [71,72].

### 4.7. Western Blotting Analysis

Five biological replicates were analyzed per group (*n* = 5 animals/group). Each biological sample was analyzed in duplicate on two separate PVDF membranes, providing two technical measurements per animal. The frontal cortex tissue was homogenized in a homogenate buffer containing 5 mM Tris-HCl and 20 mM NaCl, pH 8.0. The homogenate was centrifuged at 14,000× *g* rpm at 4 °C for 10 min. The supernatants were collected, and the cells were lysed with a lysis buffer containing 50 mM Tris-HCl, 0.15 M NaCl, 0.25% sodium deoxycholate (DOC), 0.1% sodium dodecyl sulfate (SDS), and 1% (*v/v*) protease inhibitor (1 × final concentration). The total protein concentration was measured using the BCA assay. Samples were denatured by heating at 100 °C for 5 min, and then the reaction was stopped on ice for 3 min. Subsequently, 60 µg of proteins and markers were separated by 12.50% SDS-PAGE gels under denaturing conditions and electro-transferred to a 0.45 µm polyvinylidene fluoride (PVDF) membrane.

For the detection step, membrane blocking was carried out at room temperature for 1 h using 5% non-fat milk diluted in Tris-buffered saline containing 0.10% Tween-20 (TBS-T). Following a rinse with TBS-T, membranes were incubated with primary antibodies forCalb1 (1:1000 dilution, cat #C2724, Sigma-Aldrich, St. Louis, MO, USA) for 4 h at room temperature, followed by overnight incubation at 4 °C, and anti-beta-actin (1:1000 dilution, cat #8H10D10, Cell Signaling Technology, Danvers, MA, USA) was incubated overnight at 4 °C. After three washes, membranes were incubated for 1 h with secondary antibodies: biotinylated goat anti-rabbit IgG (1:200 dilution; Cat# BA-1000, Vector Laboratories, Newark, CA, USA) for Calb1 and goat anti-mouse IgG (1:400 dilution, Cat# A-1000, Vector Laboratories, Newark, CA, USA) for beta-actin. To amplify the signal, an ABC peroxidase reagent (1:100 dilution) was applied for 60 min. Finally, Protein bands were visualized using a 3,3′,5,5′-tetramethylbenzidine (TMB) colorimetric substrate (Promega, Madison, WI, USA).

Western blot band intensities were digitized and quantified using ImageJ software (National Institutes of Health, USA). Primary normalization was carried out by dividing Calb1 density by the internal loading control (beta-actin) for each sample lane. Secondary normalization for inter-assay variability was achieved by expressing the adjusted Calb1/beta-actin values relative to the matched Control sample run on each individual membrane (defined as 1.0).

Western blot band intensities were quantified using ImageJ software (National Institutes of Health, USA). For each sample, Calb1 band intensity was normalized to the corresponding beta-actin band intensity to correct for differences in protein loading. To account for inter-membrane variability, the resulting Calb1/beta-actin ratio for each sample was further normalized to the Control sample run on the same membrane, which was assigned a value of 1.0. Each biological sample was analyzed in two technical replicates. The normalized values obtained from the two technical replicates were averaged to obtain a single value for each biological replicate for subsequent statistical analysis.

### 4.8. Statistical Analysis

Values are expressed as mean ± standard error of the mean (SEM) and were analyzed using SPSS Statistics (version 26.0), with statistical significance set at *p* < 0.05. Statistical analyses were selected according to the experimental design and specific study objectives. Baseline comparisons and outcomes measured at a single time point, including FST behavior and plasma corticosterone levels, were analyzed using one-way ANOVA followed by Dunnett’s post hoc test, where appropriate. Longitudinal measurements, including body weight and sucrose preference, were analyzed using two-way repeated-measures ANOVA, with treatment as the between-subjects factor and time as the within-subjects factor, to evaluate the main effects of treatment and time and the treatment × time interaction. Bonferroni-adjusted pairwise comparisons were performed to compare treatment groups at individual time points. For Western blot data, the Dx group was compared with the normalized Control reference value (1.0) using a one-sample *t*-test. Differences among the Dx, Dx-GOSD5, and Dx-GOSD10 groups were analyzed using one-way ANOVA followed by Dunnett’s multiple-comparison test, with the Dx group serving as the comparison group.

## Figures and Tables

**Table 1:** Animal body weights across experimental groups.

Group	Day 1	Day 7	Day 14	Day 21	Day 28
Control	286.80 ± 12.18	319.20 ± 18.47	357.40 ± 19.61	377.20 ± 18.80	390.80 ± 15.02
Control-GOSD5	291.80 ± 15.88	337.00 ± 5.05	370.20 ± 12.65	395.80 ± 12.68	410.40 ± 6.58
Control-GOSD10	292.50 ± 4.94	330.00 ± 1.58	368.00 ± 3.58	392.00 ± 4.55	397.00 ± 6.28
Dx	304.00 ± 7.34	302.80 ± 4.19	305.40 ± 3.94	259.00 ± 3.63 ***	237.60 ± 5.08 ***
Dx-GOSD5	298.00 ± 15.50	297.20 ± 9.26	304.60 ± 11.70	266.60 ± 11.25 ***	248.60 ± 10.89 ***
Dx-GOSD10	300.00 ± 11.28	293.80 ± 5.72	301.00 ± 9.06	262.00 ± 10.58 ***	242.20 ± 12.67 ***
Dx-Flu	323.80 ± 11.50	306.20 ± 7.00	287.20 ± 9.60	240.60 ± 7.45 ***	232.00 ± 7.41 ***

Values are expressed as mean ± SEM. Statistical analysis was performed using two-way repeated-measures ANOVA, with treatment as the between-subjects factor and time as the within-subjects factor, separately for baseline and Dx-induced conditions. Bonferroni-adjusted pairwise comparisons were performed at each time point. (*** *p* < 0.001 when compared with the control group).

**Table 2:** The effect of GOSD on FST, including time, latency time, and frequency behaviors.

	Time (s)	Latency (s)	Frequency
Immobility			
Control	63.78 ± 13.47	147.60 ± 11.99	11.40 ± 3.22
Control-GOSD5	37.48 ± 6.17	170.60 ± 15.15	6.60 ± 1.17
Control-GOSD10	54.51 ± 11.36	156.00 ± 13.24	9.00 ± 2.04
Dx	186.22 ± 21.35 **	31.80 ± 6.22 ***	38.80 ± 4.75 **
Dx-GOSD5	66.08 ± 4.61 ^###^	107.00 ± 16.65 ^#^	12.00 ± 1.61 ^###^
Dx-GOSD10	68.31 ± 11.58 ^##^	104.20 ± 14.41 ^#^	13.40 ± 2.38 ^##^
Dx-Flu	39.68 ± 7.96 ^###^	143.80 ± 34.03 ^##^	7.80 ± 1.32 ^###^
Swimming			
Control	212.35 ± 12.79	39.40 ± 9.45	44.00 ± 3.10
Control-GOSD5	227.56 ± 10.25	34.60 ± 9.04	46.20 ± 1.93
Control-GOSD10	215.51 ± 13.08	22.75 ± 6.91	45.00 ± 3.49
Dx	58.82 ± 13.77 ***	41.40 ± 14.74	10.60 ± 2.89 ***
Dx-GOSD5	179.24 ± 19.73 ^###^	32.60 ± 10.25	37.60 ± 3.97 ^###^
Dx-GOSD10	168.20 ± 15.45 ^##^	28.00 ± 14.11	34.60 ± 3.17 ^#^
Dx-Flu	200.97 ± 15.05 ^###^	7.40 ± 2.54	43.40 ± 3.96 ^###^
Climbing			
Control	23.32 ± 5.52	5.80 ± 1.93	4.40 ± 1.21
Control-GOSD5	31.93 ± 4.44	3.80 ± 0.73	6.60 ± 0.75
Control-GOSD10	25.53 ± 10.18	5.75 ± 2.63	5.00 ± 2.35
Dx	46.36 ± 12.74	42.20 ± 13.68 **	9.00 ± 2.51
Dx-GOSD5	53.50 ± 18.33	7.00 ± 1.05 ^##^	9.60 ± 3.50
Dx-GOSD10	53.95 ± 11.14	9.00 ± 1.87 ^##^	10.40 ± 2.14
Dx-Flu	55.65 ± 12.40	19.40 ± 4.66	9.40 ± 2.69

Values are expressed as mean ± SEM. Statistical analysis was performed by using one-way ANOVA followed by Dunnett’s post hoc test (** *p* < 0.01, *** *p* < 0.001 when compared with the Control group, # *p*< 0.05, ## *p* < 0.01, ### *p* < 0.001 when compared with the Dx group).

## Data Availability

The raw MS/MS data and accompanying analyses are publicly available in the ProteomeXchange Consortium via the jPOST partner repository under the identifier PXD082412 with the dataset identifier JPST004843 (https://repository.jpostdb.org/preview/19313098206a7bae1a8bc87, access key 8042).

## Supplementary Materials

The supplementary materials are available online at https://www.mdpi.com/article/10.3390/ijms27198721/s1.

## Abbreviations

The following abbreviations are used in this manuscript:

Atp2b4	plasma membrane calcium-transporting ATPase 4
BP	biological processes
Cacna1b	voltage-dependent N-type calcium channel subunit alpha
Cacna1g	voltage-dependent T-type calcium channel subunit alpha-1G
Calb1	calbindin
CBP	calcium-binding protein
CC	cellular component
DEPs	differentially expressed proteins
DOC	sodium deoxycholate
DTT	dithiothreitol
Dx	dexamethasone
ELISA	enzyme-linked immunosorbent assay
Flu	fluoxetine
FST	forced swimming test
GO	gamma-oryzanol
GOSD	gamma-oryzanol solid dispersion
HED	human equivalent dose
HPA	hypothalamic–pituitary–adrenal
IAA	Iodoacetamide
KEGG	Kyoto Encyclopedia of Genes and Genomes
LC-MS/MS	liquid chromatography–tandem mass spectrometry
MCAF	24-methylene cycloartanyl ferulate
MDD	major depressive disorder
MF	molecular function
Mylk	myosin light chain kinase, smooth muscle
OFT	open field test
PPI	protein–protein interaction
PVDF	polyvinylidene fluoride
PVP K30	polyvinylpyrrolidone K30
SDS	sodium dodecyl sulfate
SPT	sucrose preference test
SSRIs	selective serotonin reuptake inhibitors
TMB	3,3′,5,5′-tetramethylbenzidine
TST	tail suspension test
