# Supplementary material for: Gamma-Oryzanol Solid Dispersion Attenuates Dexamethasone-Induced Depressive-like Behaviors in Rats Associated with Calbindin-1 and Calcium Signaling
Source: Int J Mol Sci. 2026 Sep 29;27(19):8721. doi: 10.3390/ijms27198721 (PMC13664884; doi:10.3390/ijms27198721)
Supplement: Supplementary file 1 [file ijms-27-08721-s001.zip › ijms-4529416-supplementary.pdf]

Supplementary Materials

Gamma-oryzanol solid dispersion attenuates dexamethasone-induced depressive-like behaviors in rats associated with calbindin-1 and calcium signaling

2.2 Effects of GOSD on depression-related behaviors in rats

2.2.1 Open field test (OFT)

Table S1

The effect of GOSD on locomotor activity.

Group	Total distance (cm)	Number of crossings	%Time spent in central zone	%Time spent in peripheral zone
Control	714.15±97.12	52.8±80.38	11.27±1.95	88.73±1.95
Control-GOSD5	1042.15±89.39	59.60±7.16	15.13±1.54	84.87±1.54
Control-GOSD10	1258.52±172.67	66.88±10.19	19.00±4.16	81.00±3.72
Dx	892.63±138.28	66.60±9.74	13.67±4.79	86.33±4.79
Dx-GOSD5	1076.79±75.42	69.00±9.74	8.53±4.08	91.47±4.08
Dx-GOSD10	1038.11±75.42	55.2+0±10.79	5.80±1.13	94.20±1.13
Dx-Flu	937.61±153.67	72.60±11.50	4.87±1.20	95.13±1.20

Data are shown as mean ± SEM. Statistical analysis was carried out by one-way ANOVA followed by Dunnett's post hoc test

Table S2

The list of differentially expressed proteins that were upregulated in the Dx/Control and downregulated in Dx-GOSD5/Dx and Dx-GOSD10/Dx.

No.	Uniport ID	Gene name	Protein Name	Dx/Control	Dx-GOSD5 /Dx	Dx-GOSD10 /Dx
				Log2FC	Log2FC	Log2FC
1	D3ZXC1	Itga2b	Integrin subunit alpha 2b	5.1844	-3.3315	-3.9813
2	Q4V8C3	Eml1	Echinoderm microtubule-associated protein-like 1	5.0182	-2.9653	-4.5489
3	F1LW30	Unc5d	Netrin receptor	4.1421	-3.7921	-4.6111
4	F1LNS6	Ccdc146	Coiled-coil domain-containing protein 146	4.1219	-4.0608	-1.6918
5	D3ZHZ3	Osbpl3	Oxysterol-binding protein	4.0624	-4.4805	-1.9437
6	O54898	Cacna1g	Voltage-dependent T-type calcium channel subunit alpha-1G	3.5312	-6.8321	-5.9213
7	A0A0G2KAM8	Dcaf1	DDB1 and CUL4 associated factor 1	3.5148	-4.9224	-2.0626
8	E9PTK9	Ankhd1	Eukaryotic translation initiation factor 4E binding protein 3	2.7593	-1.6258	-1.8699
9	A0A0H2UHV1	Carmil3 Lrrc16b	Capping protein regulator and myosin 1 linker 3	2.7066	-2.1616	-3.0481

10	F1M775	Diaph1 Diap1	Protein diaphanous homolog 1	2.568	-1.2807	-1.6911
11	Q5FVM6	Mtmr12 Pip3ap	Myotubularin-related protein 12	2.4667	-3.4896	-1.582
12	Q811U3	Erc1 Cast2 Elks Rab6ip2	ELKS/Rab6-interacting/CAST family member 1	2.4295	-5.1625	-1.9418
13	Q810D0	Synm DMN	Desmuslin	2.4101	-3.1114	-1.7556
14	Q64599	LOC28698 7	Hemiferrin	2.3082	-5.5441	-3.6936
15	Q63563	Abcc9 Sur2	ATP-binding cassette sub-family C member 9 (Sulfonylurea receptor 2)	2.0791	-2.0889	-2.3097
16	F1LPP8	Chd3	DNA helicase	2.0686	-1.3783	-2.1825
17	F1LPF2	Agrn	Agrin	2.031	-5.9713	-1.223
18	A0A0G2K5 M7	Scaf8	SR-related CTD-associated factor 8	1.9474	-4.4775	-4.1181
19	W0NXI0	Slc4a10 NCBE	Anion exchange protein	1.8355	-5.0045	-1.5652
20	O35806	Ltbp2	Latent-transforming growth factor beta-binding protein 2	1.7794	-6.3315	-5.5515
21	Q9JJ75	Csnk1e rCK1e-3	non-specific serine/threonine protein kinase	1.7049	-4.8744	-4.6241

22	Q5QE78	Aox2 Aoh3 Aox3l1	Aldehyde oxidase 2	1.5075	-1.5154	-2.6002
23	G3V7L7	Chrna6	Cholinergic receptor nicotinic alpha 6 subunit	1.4965	-3.5882	-2.004
24	Q7TMA5	Apob	Apolipoprotein B-100	1.4467	-2.7386	-1.3245
25	Q5ZQU0	Sned1	Sushi, nidogen and EGF-like domain-containing protein 1	1.4066	-5.393	-5.4201
26	A0A0G2JTU0	Arhgef15	Rho guanine nucleotide exchange factor 15	1.3343	-2.4174	-2.2506
27	P34058	Hsp90ab1 Hsp84 Hspcb	Heat shock protein HSP 90-beta	1.2463	-3.8146	-6.1795
28	P27008	Parp1 Adprt	ADP-ribosyltransferase diphtheria toxin-like 1	1.2217	-3.3058	-4.024
29	A0A140TA94	Chrd	Chordin	1.2162	-2.2189	-1.6415

Table S3

The list of differentially expressed proteins that were downregulated in the Dx/Control and upregulated in Dx-GOSD5/Dx and Dx-GOSD10/Dx.

No.	Uniport ID	Gene name	Protein Name	Dx/Control Log2FC	Dx-GOSD5 /Dx Log2FC	Dx-GOSD10 /Dx Log2FC
1	Q5EB94	Myzap Myozap	Myocardial zonula adherens protein	-1.3298	3.2662	5.6911
2	D4A055	Cacnb4	Voltage-dependent L-type calcium channel subunit beta-4	-1.4357	3.5485	2.1983
3	Q99NB7	Acot12 Cach Cach1	Acetyl-coenzyme A thioesterase	-1.5558	1.2816	1.8785
4	D3ZG74	Frem2	FRAS1 related extracellular matrix 2	-1.5877	2.6479	2.5591
5	B4F7B5	RGD1564456	RGD1564456 protein	-1.6269	4.5926	7.7721
6	F1LVC2	Vom2r30 AABR07002473.1	Vomeronasal 2 receptor, 30	-1.6411	1.5028	2.0178
7	F1LWZ8	Lemd3	LEM domain containing 3	-1.6513	1.4574	1.4113

8	O55164	Mpdz Mupp1	Multiple PDZ domain protein	-1.6971	1.1276	1.569
9	Q811A3	Plod2	Procollagen-lysine,2-oxoglutarate 5-dioxygenase 2	-1.8531	2.8352	1.7547
10	D4A065	Adamts6	ADAM metallopeptidase with thrombospondin type 1 motif, 6	-1.9024	4.4371	2.2787
11	Q63425	Prx	Periaxin	-1.9428	1.8582	2.3866
12	D3ZSG2	Trank1	Tetratricopeptide repeat and ankyrin repeat containing 1	-1.9449	1.2691	1.9147
13	A0A0G2K4D0	Ank3	Ankyrin-3	-2.1323	1.9716	2.89
14	A0A0G2JX32	Amph LOC100909679 LOC100910792	Amphiphysin-like	-2.194	3.9577	5.4435
15	A0A0G2JYZ1	Ttbk2	Tau tubulin kinase 2	-2.2463	2.1383	2.646
16	D4A6P3	Shtn1 RGD1311558	Shootin-1	-2.6672	3.1114	3.7631
17	Q8K4K5	Llgl1 Rgl1	Lethal(2) giant larvae protein homolog 1	-2.7027	4.3316	5.1207
18	A0A0G2QC49	Zfp951	Zinc finger protein 951	-2.7658	1.8902	1.5476

19	P35570	Irs1 Irs-1	Insulin receptor substrate 1	-2.8249	4.6183	5.7324
20	Q63272	Jak3	Tyrosine-protein kinase JAK3	-2.8361	3.2629	2.1414
21	G3V6S2	Aco1	Cytoplasmic aconitate hydratase	-3.1414	3.6015	2.9001
22	Q3KR84	4931429L15Rikl LOC500990	Similar to RIKEN cDNA 4931429L15	-3.1761	5.092	5.4336
23	A0A096MK54	Synm	Synemin	-3.1807	3.0247	3.1282
24	P05370	G6pdx G6pd	Glucose-6-phosphate 1-dehydrogenase	-3.2188	2.5548	2.27
25	F1LNR0	Clasp1	Cytoplasmic linker associated protein 1	-3.2842	3.4249	4.5048
26	A0A0G2K5V8	Dapk1	non-specific serine/threonine protein kinase	-3.3091	2.4098	2.9323
27	Q0D2L7	Cchcr1 Hcr	Coiled-coil alpha-helical rod protein 1	-3.342	4.6945	4.4718
28	F1LPC8	Ntn1	Netrin-1	-3.3579	1.5663	1.7867
29	A0A0G2JT74	Fam149b1	Family with sequence similarity 149, member B1	-3.4474	3.4359	4.9413
30	D4A346	Acap3	Arf-GAP with coiled-coil, ANK repeat and PH domain-containing protein	-3.4933	3.4335	5.6301
31	D3ZFU9	Mylk	Myosin light chain kinase, smooth muscle	-3.5642	3.5601	3.402

32	D4A5B3	C2cd5	C2 calcium-dependent domain containing 5	-3.5832	4.6645	2.3345
33	A0A0G2K5B6	Ambra1	Autophagy and beclin 1 regulator 1	-3.6233	2.6456	3.2445
34	P47858	Pfkm Pfk-m	ATP-dependent 6-phosphofructokinase	-3.6447	3.5086	4.7083
35	A0A0G2JW65	Rasgrf2 AABR07007642.1	RAS protein-specific guanine nucleotide-releasing factor 2	-3.6509	1.9225	3.734
36	P12847	Myh3	Myosin-3	-3.7031	3.5952	7.6201
37	Q9WUI9	Ncoa2 Tif2	Nuclear receptor coactivator 2	-3.7139	2.5796	7.5934
38	Q62824	Exoc4 Sec8 Sec8l1	Exocyst complex component 4	-3.7311	2.2557	6.6678
39	Q8VIE2	Pde7b RNPDE7B	Phosphodiesterase	-3.8031	3.1523	3.1119
40	Q9Z221	Pmfbp1 Odf3	Polyamine-modulated factor 1-binding protein 1	-3.871	4.4475	4.4247
41	D4A7C2	Fmn1	Formin 1	-3.9086	2.8382	4.9183
42	Q6S3A0	Plec	Plectin 6	-3.9788	1.8248	1.7049
43	D4A915	Ano1	Anoctamin	-4.0023	3.0431	4.6477
44	D3ZD24	Syne3	Spectrin repeat containing, nuclear envelope family member 3	-4.1622	3.4256	1.9517
45	A0A0H2UHX6	Ica1l	Islet cell autoantigen 1-like	-4.1832	4.3649	1.8289

46	Q62918	Nell2 Nel	Protein kinase C-binding protein NELL2	-4.1889	1.2761	4.8591
47	A0A0G2K2V5	Exoc1	Exocyst complex component 1	-4.2275	1.9901	2.2776
48	Q63312	Phldb1 Ll5	Pleckstrin homology-like domain family B member 1	-4.2908	2.8495	3.8465
49	F1LQJ7	Pck2	phosphoenolpyruvate carboxykinase (GTP)	-4.5049	4.0174	3.7633
50	Q80UE2	Cyp1a1 CYP450IAI	Cytochrome P450 1A	-4.6148	7.1903	7.7175
51	F1M048	Cux2	Homeobox protein cut-like	-4.6915	1.8787	3.1484
52	D3ZEZ9	Svil	Supervillin	-4.77	2.4189	3.1905
53	Q62888	Nlgn2	Neuroligin-2	-4.7919	4.4068	5.1549
54	M0RD44	Zfp318	Zinc finger protein 318	-4.9247	3.1504	4.0994
55	P15800	Lamb2	Laminin subunit beta-2	-5.0815	3.1255	3.1632
56	Q6WDI9	Rag1 RAG1	V(D)J recombination-activating protein 1	-5.1006	4.0039	5.5666
57	D3ZW40	Mtmr4	protein-tyrosine-phosphatase	-5.2966	2.9885	2.9569
58	A0A0G2K665	Tgap1 tGap1	GTPase activating protein testicular GAP1	-5.5996	5.3916	2.5249
59	G3V6Q9	Brinp1 Dbc1	BMP/retinoic acid-inducible neural-specific protein 1	-5.7225	4.9522	7.4745

60	Q8VGC3	Cacnb2	Voltage-dependent L-type calcium channel subunit beta-2	-5.7514	1.597	5.8545
61	F1M1F5	Megf11	Multiple EGF-like-domains 11	-5.8054	5.3477	3.4499
62	F1M7U2	Capn11	Calpain 11	-6.0062	4.4356	3.5171
63	F1LQ87	Cacna1b	Voltage-dependent N-type calcium channel subunit alpha	-6.0062	2.2923 +	2.874
64	Q64542	Atp2b4	Plasma membrane calcium-transporting ATPase 4	-6.039	2.2923	4.0923
65	Q6QMG5	Abcc4	Multidrug resistance-associated protein 4 splice variant	-6.0707	3.9259	3.9486
66	D3ZMH5	Tiparp	TCDD-inducible poly(ADP-ribose) polymerase	-6.1827	3.1233	1.6975
67	Q6UPE2		Cardiotrophin-like cytokine	-6.2308	4.9021	7.1684
68	Q3ZU82	Golga5	Golgin subfamily A member 5	-6.2827	10.285 3	4.0428
69	P97629	Lnpep	Leucyl-cystinyl aminopeptidase	-6.7614	5.9621	4.476
70	P51870	Cyp4f5	Cytochrome P450 4F5	-6.8322	3.939	5.3468

2.4.5 Validation of calcium-binding protein Calb1 expression

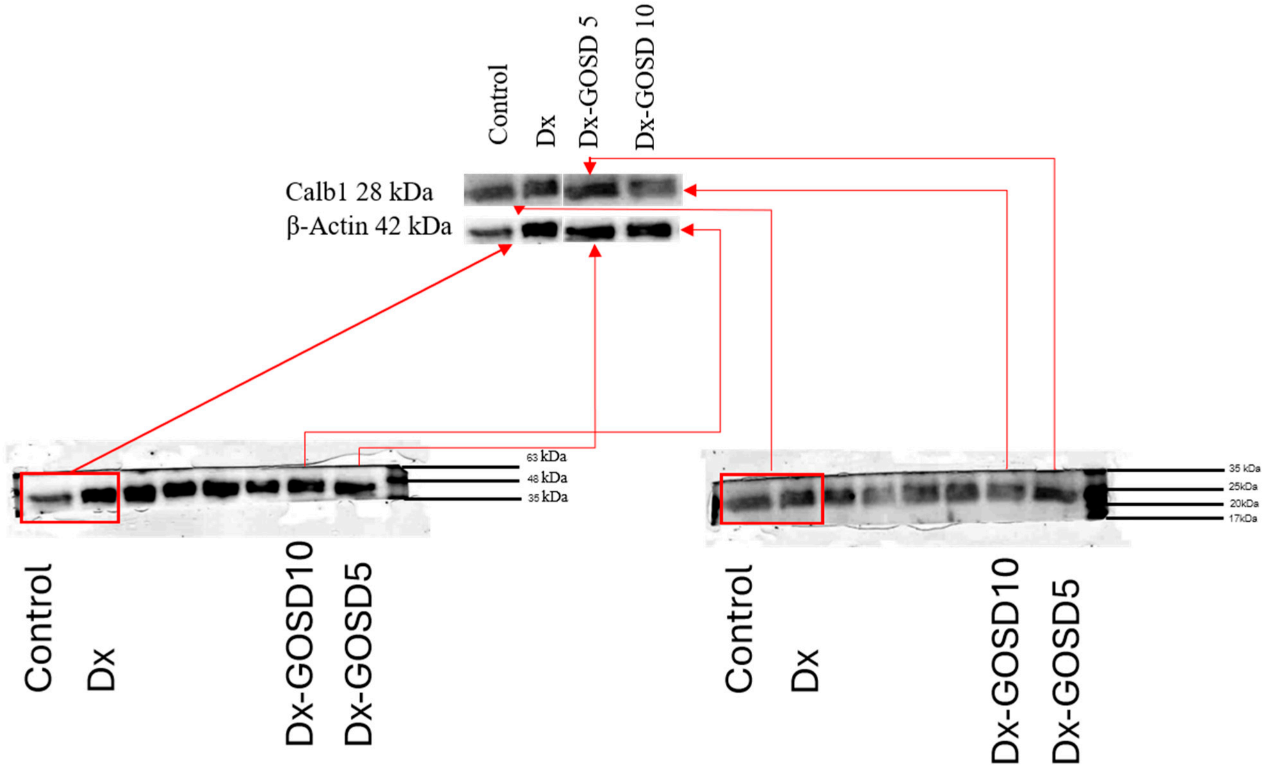

Figure S1. Full-length blots corresponding to Figure 6. Lane 1: Control group; Lane 2: Dx group; Lanes 7: Dx-GOSD10 group; Lane 8: Dx-GOSD5 group.
